# Pd/SiC-Catalyzed Visible-Light-Driven N-Methylation of Nitroaranes Using Formaldehyde

**DOI:** 10.3390/nano15181394

**Published:** 2025-09-10

**Authors:** Dongfang Hou, Ruifeng Guo, Xianshu Dong, Yuping Fan, Jingru Wang, Xili Tong

**Affiliations:** 1 Center for Analysis Testing and Equipment Sharing of TYUT, Taiyuan University of Technology, Taiyuan 030024, China; 2 College of Mining Engineering, Taiyuan University of Technology, Taiyuan 030024, China; 3 State Key Laboratory of Coal Conversion, Institute of Coal Chemistry, Chinese Academy of Sciences, Taiyuan 030001, China

**Keywords:** SiC, nitro compounds, methylation, photocatalysis

## Abstract

Pd nanoparticles (Pd/SiC) with a main exposed plane of Pd (111) were prepared by liquid phase reduction. The use of formaldehyde as a methylation reagent for the photocatalytic methylation of aromatic nitro compounds to N,N-methylaniline resulted in one-pot methylations of aromatic nitro compounds with high photocatalytic activity and selectivity under mild reaction conditions. The high catalytic activity of Pd/SiC in N-methylation reactions arises from the Mott–Schottky contact between Pd and SiC, which promotes the transfer of photogenerated electrons to Pd. The high selectivity is ascribed to the ability of Pd nanoparticles to catalyze the hydrogenation of nitro groups to amino groups, which subsequently undergo direct methylation with formaldehyde, bypassing the intermediate formylation step.

## 1. Introduction

N-methylated amines are an important part of the synthesis of a range of valuable compounds, including dyes, surfactants, drugs, preservatives, and agrochemicals [1].

The reduction in inexpensive and readily available nitroaromatics is one of the most common methods for the synthesis of aniline. Since the nitro group contains two oxygen atoms, substitution of one oxygen atom with a methyl group leads to the formation of N-methylaniline, while further methylation yields N,N-dimethylaniline. Both compounds serve as important chemical intermediates [2,3]. Consequently, one-pot synthesis approaches enabling the direct hydrogenation of nitroaromatics to N-methylated amines have garnered significant attention in recent years [4].

Traditional N-methylation methods typically employ sulfuric acid as a catalyst; however, these processes require high temperatures and substantial amounts of alkali, generate considerable waste, and often suffer from low selectivity and yield [5]. Therefore, the design and preparation of high-performance catalysts and the development of new N-methylation methods to meet the needs of green environmental protection have become important development directions in this field.

The N-methylation reactions initially used homogeneous catalysts, such as Ir [6,7,8], Ru [1,9,10,11,12], Rh [13], Mn [14,15], and Fe [16], to prepare N-monomethylated products by reacting aniline with methanol. For instance, Maji et al. reported that homogeneous Ru catalysts can efficiently catalyze the N-methylation of aromatic amines and the N,N-dimethylation of aliphatic amines [12]. Similarly, Ogata et al. demonstrated that homogeneous Ru catalysts enable the selective synthesis of N-monomethylated aliphatic primary amines, with high selectivity achieved by employing H_2_ as a reductant at lower reaction temperatures, effectively suppressing the dehydrogenation of methanol [9]. Although various homogeneous catalysts have shown effectiveness in N-methylation, their practical application is limited by the need for specific ligands, high sensitivity to moisture and air, and challenges in separation. In contrast, heterogeneous catalysts offer significant advantages, including ease of storage, straightforward separation, and recyclability, making them particularly attractive for industrial applications. Consequently, the development of new heterogeneous catalysts with high selectivity and activity has become a major focus of current research.

In recent years, N-methylation reactions catalyzed by heterogeneous catalysts have been reported frequently. For example, Xu et al. reported a method for synthesizing N,N-dimethyl aniline from nitrobenzene using a Raney nickel catalyst at 170 °C and 3 MPa of N_2_ pressure [17]. Uozumi and Sakurai achieved N-methylation using CuAlO_x_ catalysts and CO_2_/H_2_ [18]. And then, an active Pd/CuZrO_x_ catalyst was found for the reductive amination of CO_2_ under mild reaction conditions, the N-methylation of amines and nitro compounds with CO_2_/H_2_ can be realized with up to 97% yield [19]. Zhang et al. used a Pd (8 mol%)/TiO_2_ nanocatalyst to perform a similar transformation process under ultraviolet light [20]. However, these reactions require higher pressures (30–70 bar) and temperatures (170 °C) as well as longer reaction times (48 h). Moreover, current studies on amine methylation predominantly employ pre-synthesized amine compounds as substrates, achieving methylation through the condensation of amines with CO_2_ or formaldehyde (e.g., Mannich reaction) combined with catalytic hydrogenation. The selectivity of monomethyl and dimethyl products is poor, and only specific substrates can be catalyzed.

Photocatalytic organic synthesis provides a new channel for the specific conversion of organic substances into products; in particular, visible light photocatalytic reactions have mild conditions, which can effectively improve the reaction efficiency and reduce the waste of resources. As a stable semiconductor material, SiC exhibits strong visible-light absorption capabilities [21,22,23]. Pd nanocatalysts supported on SiC have demonstrated excellent photocatalytic activity and selectivity in various hydrogenation reactions, including the hydrogenation of furan [24], nitrobenzene [25], and benzonitrile [26] to produce amine compounds. Moreover, palladium nanoparticles, similar to metal nanoparticles such as Au, Ag, and Cu, are capable of exhibiting localized surface plasmon resonance (LSPR) effects [27]. Although the LSPR effect of palladium is relatively weak in the visible light region, it is particularly pronounced in the ultraviolet spectral range. This is primarily attributed to the characteristics of palladium’s electronic band structure, where interband transitions exhibit strong absorption capacity for high-energy photons in the ultraviolet and visible light regions [28]. Cai et al. have reported that under room-temperature reaction conditions, light irradiation can significantly enhance the intrinsic catalytic performance of Pd [29]. Therefore, Pd-based nanocatalysts have shown excellent activity and selectivity in many catalytic conversion reactions [30].

In this work, a Pd/SiC photocatalyst was synthesized via a liquid-phase reduction method. Under mild reaction conditions (40 °C, 1 atm H_2_, and 0.25 W/cm^2^ Xe lamp irradiation), employing nitro compounds as the starting substrate, a one-pot simultaneous “nitro reduction” and “in situ methylation of the resulting amine” were achieved within a single system to produce N,N-dimethylaniline using formaldehyde with high selectivity. This approach offers a novel route for the direct construction of C-N bonds and the introduction of methyl groups. The method is also applicable to aromatic nitro compounds with various electron-withdrawing and electron-donating substituents. In the Pd/SiC catalyst, SiC demonstrates broad-spectrum light absorption and efficient photothermal conversion, enabling excellent photocatalytic activity under visible light irradiation. And a chemically inert surface that minimizes non-specific adsorption and unwanted side reactions. Furthermore, the formation of a Mott–Schottky junction at the Pd/SiC interface under light irradiation drives efficient electron transfer to Pd, enriching its electron density and thereby enhancing the nucleophilic addition of formaldehyde to the amino intermediates.

## 2. Experimental Section

### 2.1. Preparation of Catalysts

The Pd/SiC catalyst was prepared using the liquid-phase reduction method [25], the preparation steps are as follows: First, 970 mg of SiC was dispersed in 28.2 mL of a 0.01 M Pd (NO_3_)_2_ solution and stirred at 300 rpm for 30 min. Next, 20 mL of a 0.53 M lysine aqueous solution was added dropwise to the mixture under stirring for 30 min. The stirring rate was then increased to 800 rpm, and 10 mL of freshly prepared 0.35 M NaBH_4_ aqueous solution was added dropwise in 20 min, turning the mixture from light green to dark blue. After this, 10 mL of 0.3 M hydrochloric acid was added, and the mixture was stirred for 24 h. The catalyst was then washed three times with water and ethanol. The separated catalyst is dried in a vacuum oven at 60 °C for 12 h to obtain the Pd/SiC catalyst with a palladium loading of 3 wt%. Catalysts with varying Pd loadings can be prepared by adjusting the amount of Pd (NO_3_)_2_ used. 3 wt% Pd/TiO_2_ and Pd/Al_2_O_3_ catalysts were also synthesized using a similar method.

### 2.2. Catalyst Characterization

The morphology and structure of the catalyst were analyzed using high-resolution transmission electron microscopy (HRTEM, JEM-2100F) (JEOL, Tokyo, Japan). The crystal structure of the catalyst was determined by X-ray diffraction (XRD, Rigaku D-Max/RB, Tokyo, Japan). X-ray photoelectron spectroscopy (XPS) was used to analyze the changes in the content and valence state of the Pd metal in the catalyst using Al Kα (hν = 1486.6 eV) X-ray line on a Kratos XSAM800 spectrometer (Shimadzu/Kratos, Manchester, UK). The light absorption characteristics of the catalyst were tested using a UV-visible absorption spectrometer (UV-vis, UV-3600, Shimadzu, Japan) with Al_2_O_3_ as the reference. The efficiency of photogenerated electron–hole separation in the catalyst was evaluated using photoluminescence spectroscopy (PL, F-7000, Hitachi, Japan) under a 320 nm excitation wavelength (with a 390 nm filter) at room temperature. The carrier separation efficiency and the lifetime of charge carriers were further analyzed by time-resolved photoluminescence spectroscopy (TR-PL) on a fluorescence spectrum (FLS1000, Edinburgh Instruments Ltd., Livingston, UK) with an excitation wavelength of 365 nm and a test wavelength of 460 nm. The optical absorption properties were determined using a UV-Vis-NIR spectrophotometer (UV-2600i DH7001, Shimadzu, Japan) by performing diffuse reflectance spectroscopy on SiC samples with a BaSO_4_ standard white plate. The band gap energy (Eg) was determined from the Tauc plot by extrapolating the linear region of the (αhν)^2^ vs. hν plot, where α is the absorption coefficient, h is Planck’s constant, and ν is the light frequency.

### 2.3. Catalytic Reaction

The photocatalytic reaction system was conducted in a sealed high-pressure reactor made of transparent quartz glass. The system consists of 30 mg of catalyst, 7 mL of anhydrous ethanol as the solvent, 0.8 mmol of 2-nitrobenzyl ether (2-NA) or other aromatic nitro compounds, and 0.13 mL of a 37% formaldehyde solution. The reaction conditions were as follows: 1 atm H_2_, 40 °C, 600 rpm, and exposure to xenon light for 3 h. The xenon lamp had an intensity of 0.25 W/cm^2^ and an output wavelength of 400–800 nm. The light intensity can be adjusted to investigate its effect on the reaction. After the reaction, 2 mL of the mixture was centrifuged and filtered through a microporous filter (0.22 μm) to remove solid catalyst particles. The filtrate was then analyzed using gas chromatography-mass spectrometry (GC-MS, Bruker SCION SQ456, Bremen, Germany) in selective ion monitoring (SIM) mode to measure changes in the concentrations of the reactants and products. The conversion rate, selectivity, and yield are calculated using Formulas (1)–(3).

(1) $$\mathrm{Conv}.\;(\%)=\frac{M_{0,\;\mathrm{reactants}}-M_{t,\;\mathrm{reactants}}}{M_{0,\;\mathrm{reactants}}}\times 100\%$$

(2) $$\mathrm{Select}.\;(\%)=\frac{M_{t,\;\mathrm{products}}}{M_{0,\;\mathrm{reactants}}-M_{t,\mathrm{reactants}}}\times 100\%$$

(3) Yield (%)=Conv.×Select.×100%

## 3. Results and Discussion

### 3.1. Synthesis and Characterization

**Figure 1:**
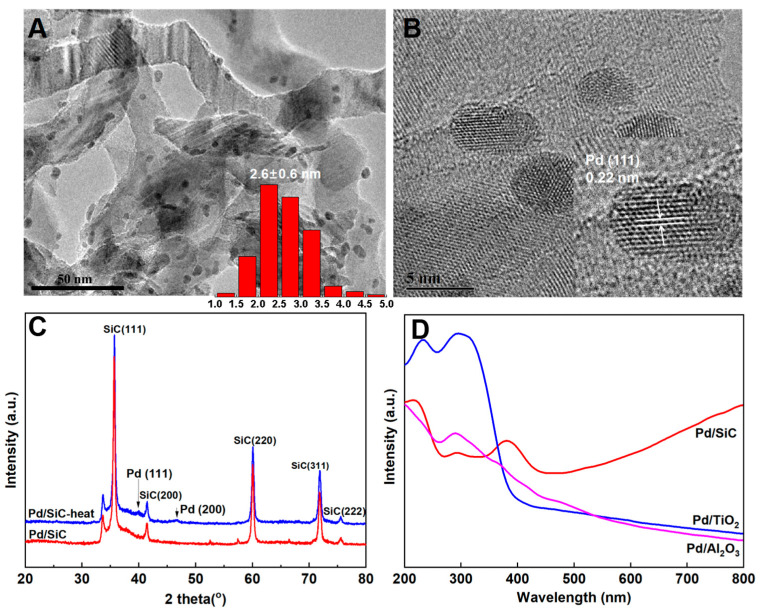
Structural and optical properties of the supported Pd catalysts. (**A**,**B**) TEM images of the 3 wt% Pd/SiC catalyst with insets showing the particle size distribution of Pd particles (**A**) and an HRTEM image (**B**); (**C**) XRD patterns of the Pd/SiC catalyst before and after thermal treatment at 400 °C; (**D**) UV-vis absorption spectra of the 3 wt% Pd/SiC, Pd/TiO_2_, and Pd/Al_2_O_3_ catalysts.

Figure 1A,B show the TEM images of the 3 wt% Pd/SiC catalyst, with the insets displaying the particle size distribution of Pd nanoparticles and the corresponding HRTEM image. Figure 1A,B clearly illustrate the morphology of the Pd/SiC catalyst and the size of the metal particles. The Pd nanoparticles are uniformly dispersed in a spherical form across the surface of the SiC support. Based on the analysis of over 300 nanoparticles using Nano Measurer software (Image J), the average particle size of Pd is determined to be 2.6 nm. The HRTEM image reveals a lattice spacing of 0.22 nm, which corresponds to the (111) crystal plane of metallic Pd [31].

Figure 1C shows the XRD pattern of Pd/SiC. The five prominent diffraction peaks correspond to the characteristic diffraction peaks of β-SiC. For Pd/SiC, no diffraction peaks of Pd are observed, which is due to the small size of the Pd nanoparticles and their high dispersion on the SiC surface. After high-temperature treatment at 400 °C for 2 h in an inert atmosphere, the Pd/SiC-heat catalyst exhibited diffraction peaks at 2θ = 40.1° and 2θ = 46.7°, corresponding to the Pd (111) and Pd (200), respectively. Patrizia Canton et al. investigated Pd nanoparticles under thermal treatment and observed alterations in their size and structure. High-temperature processing promoted the aggregation and growth of Pd nanoparticles [32]. In the Pd/SiC catalyst examined in this study, the high-temperature environment provided sufficient energy for the initially highly dispersed and small-sized Pd nanoparticles to overcome interparticle energy barriers, initiating their mutual approach and aggregation. As aggregation progressed, the particle size gradually increased, resulting in the emergence of Pd diffraction peaks in the XRD patterns that were previously undetectable due to the small size and high dispersion of the particles. Simultaneously, the aggregation and growth process led to changes in the crystal orientation of the nanoparticles. Simultaneously, during the aggregation and growth process of Pd nanoparticles, there is a change in crystal structure orientation. The (200) crystal planes that were originally less exposed are now more readily exposed, resulting in corresponding diffraction peaks appearing in the XRD spectrum.

Figure 1D displays the UV-visible absorption spectra of Pd/SiC, Pd/TiO_2_, and Pd/Al_2_O_3_. Among these, Pd/SiC exhibits significantly higher absorption intensity in the visible light region compared to Pd/TiO_2_ and Pd/Al_2_O_3_. Pd/TiO_2_ demonstrates superior absorption in the ultraviolet region below 400 nm. Notably, Pd/SiC shows a pronounced absorption peak at 375 nm, consistent with previously reported UV-visible absorption characteristics of Pd/SiC [22]. The absorption spectra of Pd/SiC show an increase above 500 nm due to the strong absorption of infrared light by SiC and the reflection from the sample during processing.

**Figure 2:**
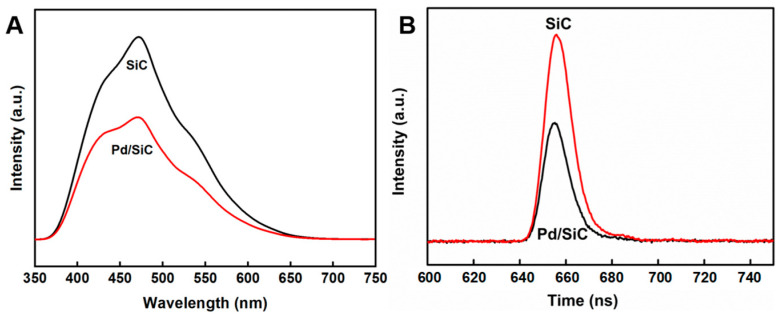
Photoluminescence spectra of pure SiC and Pd/SiC (excitation wavelength, 320 nm) (**A**), time-resolved transient decay (TR-PL) spectra at a 450 nm emission wavelength (**B**).

The separation efficiency of photogenerated electron–hole pairs in the Pd/SiC catalyst during the photochemical reaction was assessed using steady-state and time-resolved fluorescence spectroscopy [33,34,35]. Figure 2A,B present the fluorescence spectra and time-resolved transient fluorescence spectra of Pd/SiC, respectively. As shown in Figure 2A, Pd/SiC exhibits significantly lower fluorescence intensity compared to pure SiC, suggesting that the incorporation of Pd nanoparticles effectively enhances the separation of photogenerated charge carriers. This enhancement is attributed to the formation of a Mott–Schottky contact between Pd and semiconductor SiC, which facilitates the separation of electron–hole pairs under light illumination [36]. Time-resolved transient fluorescence measurements (Figure 2B) further reveal that the fluorescence lifetimes of SiC and Pd/SiC are 6.36 ns and 5.38 ns, respectively, indicating efficient charge transfer dynamics in the Pd/SiC system. The reduced carrier lifetime indicates rapid charge transfer, significantly enhancing the separation efficiency. This result further demonstrated the effective suppression of photogenerated electron–hole recombination in Pd/SiC.

**Figure 3:**
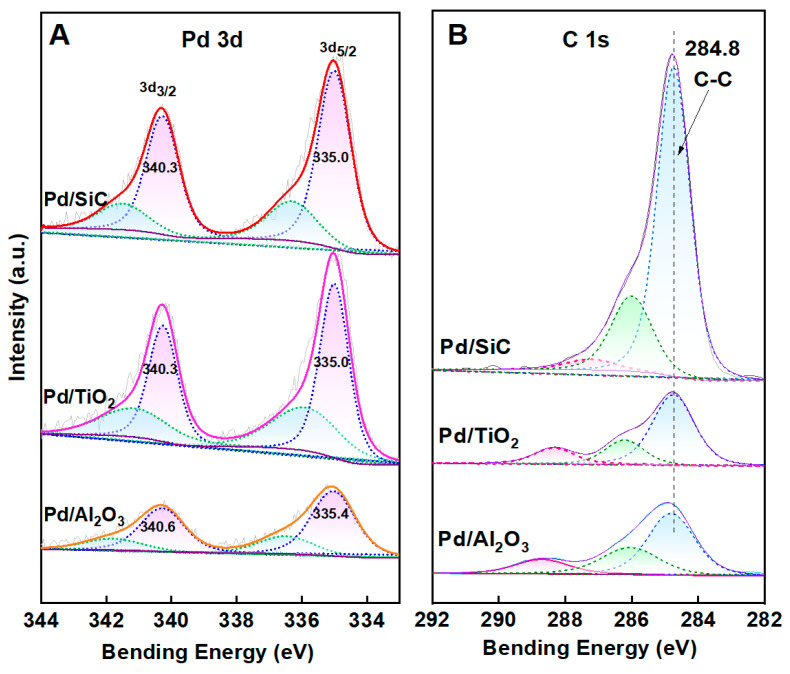
XPS spectra of the Pd/SiC, Pd/TiO_2_, and Pd/Al_2_O_3_. (**A**) Pd 3d and (**B**) C 1s. The C 1s signals in Pd/TiO_2_, and Pd/Al_2_O_3_ are attributed to adventitious carbon used for binding energy calibration.

Figure 3 displays the XPS results for Pd/TiO_2_, Pd/Al_2_O_3_, and Pd/SiC catalysts. Analysis of the Pd 3d (Figure 3A) orbital states confirms that palladium predominantly exists in its metallic state (Pd^0^) across all three catalysts. Notably, the peak intensities for Pd/TiO_2_ and Pd/SiC are higher than those for Pd/Al_2_O_3_, suggesting superior dispersion of Pd nanoparticles on TiO_2_ and SiC supports. Figure 3B are C regions of the Pd/TiO_2_, Pd/Al_2_O_3_, and Pd/SiC. They display three peaks. The peak at 284.8 eV can be attributed to the c1s binding energy of carbon. There are also two peaks at the binding energy higher than 286 eV, which can be attributed to the mixture of the oxidized forms of carbon, namely other carbon oxides [22]. In the case of Pd/TiO_2_ and Pd/SiC, the binding energy of Pd (Pd 3d_5/2_ ≈ 335.0 eV) is slightly lower than that of pure Pd particles (335.4 eV), indicating an electron-rich surface of Pd. This is because both the TiO_2_ and SiC supports are semiconductors with work functions (TiO_2_ is 4.51–5.62 eV [37], SiC is 4.0 eV [38]) lower than that of metallic Pd (5.12 eV). This work function difference promotes the formation of Mott–Schottky contacts between the semiconductors and the metal, driving electron transfer from the semiconductor to the metallic region, thereby increasing the electron density on Pd and stabilizing its metallic state against oxidation by atmospheric oxygen.

### 3.2. Photocatalytic Performance of the Catalyst

**Figure 4:**
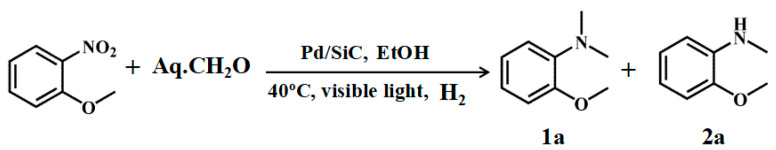
Photocatalytic reductive N-methylation of 2-nitroanisole using Pd/SiC.

Using 2-nitrobenzyl ether (2-NA) and formaldehyde solution N-methylation reactions as probe reactions (see Figure 4), the photocatalytic activity of the catalyst was investigated (see Table 1). When SiC is used as the catalyst, the reactants do not undergo significant transformation under both light and dark conditions, indicating that SiC lacks catalytic activity in this reaction system. Under identical reaction conditions, Pd/SiC exhibits remarkable photocatalytic activity and selectivity. Specifically, at 40 °C, 1 atm H_2_, and a light intensity of 0.25 W/cm^2^ over a reaction period of 3 h, the conversion rate of 2-nitroanisole (2-NA) reaches 95%, with a selectivity of 94.2% toward 2-methoxy-N,N-dimethylaniline (**1a**). The remaining product is identified as 2-methoxy-N-methylaniline (**2a**). The Pd/SiC catalytic system achieved an AQY of 8.4% at a wavelength of 470 nm. In the absence of light, the conversion rate drops substantially to 46.1%, underscoring the critical role of light in enhancing the catalytic efficiency. Furthermore, when H_2_ is replaced by Ar in the reaction system, no reaction is observed, confirming that H_2_ serves as the sole hydrogen source in this transformation.

To evaluate whether other metal nanoparticles supported on SiC exhibit comparable catalytic performance, Pt/SiC and Ru/SiC catalysts were synthesized, with their morphology and particle size characterized as shown in Figure S1. Under identical reaction conditions, Pt/SiC displayed superior photocatalytic activity relative to Pd/SiC; however, it exhibited lower product selectivity. Despite its smaller particle size and uniform dispersion on the SiC surface, Ru showed very poor activity for the methylation reaction, with virtually no conversion. A comparison of the effects of different supports on the reaction revealed that the activities of Pd/Al_2_O_3_, Pd/TiO_2_, and Pd/ZrO_2_ were significantly lower than that of Pd/SiC. However, the selectivity for 2-methoxy-N,N-dimethyl aniline was greater than 90%, indicating that Pd nanoparticles are the primary factor in achieving high selectivity. By adjusting the amount of Pd (NO_3_)_2_, 1 wt% and 5 wt% Pd/SiC were prepared using the same method. The particle sizes of Pd nanoparticles in the two catalysts were measured to be 2.3 nm and 2.6 nm, respectively, as shown in Figure S2. When the total Pd content in the reaction system was held constant, the catalytic performance of these materials in the N-methylation reaction was inferior to that of the 3 wt% Pd/SiC catalyst. These results suggest that only an optimal Pd loading can effectively facilitate the N-methylation reaction, ensuring both high activity and selectivity.

**Figure 5:**
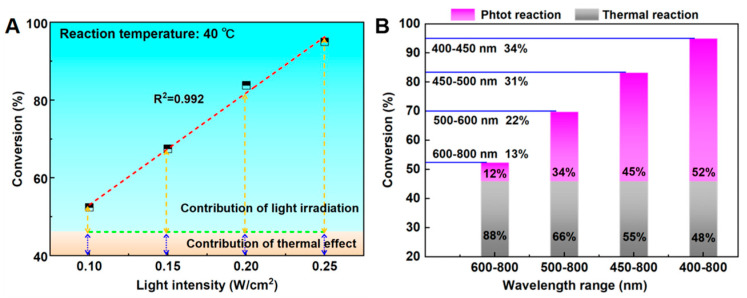
Dependences of the catalytic activity of Pd/SiC for the N-methylation reaction on the intensity (**A**) and wavelength (**B**) of irradiation.

Since light significantly enhances the reaction activity, all other reaction conditions were kept constant except for the light intensity to investigate its effect on the reaction (Figure 5A). The contribution of light to the overall conversion rate was determined by subtracting the conversion rate observed under dark conditions from that measured under illumination. The dark reaction rate was attributed solely to thermal effects. As shown in Figure 5A, increasing light intensity leads to an approximately linear increase in the conversion rate of 2-NA. Furthermore, the relative contribution of light becomes more pronounced at higher intensities: at 0.10 W/cm^2^, the light contribution is 12%, whereas at 0.25 W/cm^2^, it rises to 51.5%. This enhancement is attributed to the generation of a greater number of high-energy electrons under stronger illumination, which effectively drive the reaction [39]. Additionally, intense light induces a localized electromagnetic field around the Pd nanoparticles—a phenomenon known as the field enhancement effect—which strengthens the interaction between Pd nanoparticles and reactant molecules, thereby augmenting the catalytic efficiency of the N-methylation reaction.

To investigate the dependence of the catalytic activity of N-methylation reactions on light wavelength (see Figure 5B), a series of optical cutoff filters were used to allow light within specific wavelength ranges to reach the reaction system. In each specific wavelength region, all light intensities are maintained at 0.25 W/cm^2^. Under the full wavelength range, the conversion rate of 2-NA can reach 95%. When a 450 nm filter was used, the conversion rate decreased to 78.2%. Similarly, under light with wavelengths ranging from 500 to 800 nm and 600 to 800 nm, the conversion rates of 2-NA are 62.9% and 52.3%, respectively. By subtracting the contribution from thermal reactions, the relative contributions of different wavelength ranges to the overall photocatalytic activity were determined. The contribution rates for the 400–450 nm, 450–500 nm, 500–600 nm, and 600–800 nm ranges were calculated as 34%, 31%, 22%, and 13%, respectively. Among these, the 400–450 nm range contributed the most significantly, attributable to the strong optical absorption of Pd/SiC in wavelengths below 460 nm. In general, shorter-wavelength light possesses higher photon energy, which facilitates the excitation of reactant molecules on the Pd surface by elevating Pd to higher energy states. Conversely, light in the 600–800 nm range contributed only 13%, indicating that infrared radiation exerts a relatively minor influence on the reaction process.

### 3.3. Versatility of Catalysts

To investigate the reaction scope of Pd/SiC-catalyzed N-methylation reactions, aromatic nitro compounds with various substituents were used as substrates, as shown in Table 2. Pd/SiC has high activity and selectivity for electron-donating groups such as methyl, amino, hydroxyl, and ether groups (entries 2–5). Electron-withdrawing groups such as ketones and aldehydes have excellent activity and selectivity (entries 6–7), although they perform poorly with cyano and halogenated groups. In the case of 4-nitrobenzamide, after 5 h of reaction, the primary product is 4-methylaminobenzamide, which is N-methylated, with a selectivity of 61.3%. The remaining products are N,N-dimethyl derivatives, notably 4-dimethylaminobenzamide. For nitro compounds containing halogens, dehalogenation occurs during the reaction, yielding aniline and formaldehyde as primary intermediates, which subsequently undergo methylation—similar to the pathway observed for nitrobenzene. The decreased selectivity observed with halogenated nitro compounds is attributed to the dehalogenation that takes place during the hydrogenation step, converting nitro groups to aniline.

### 3.4. Stability of the Catalystsen

**Figure 6:**
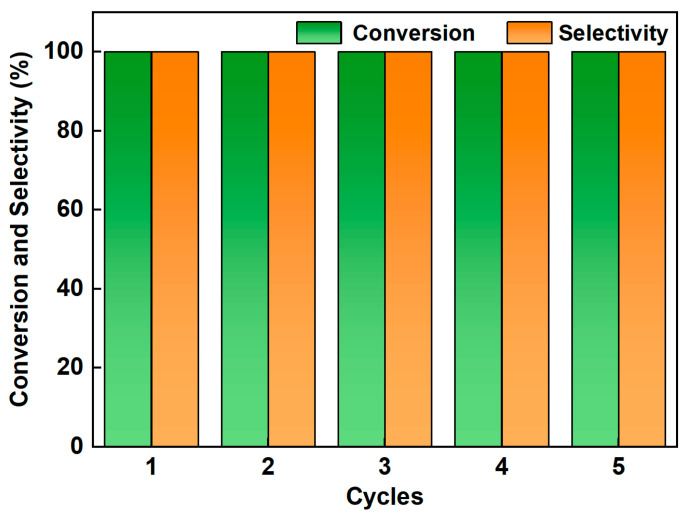
Recycling performance of 3 wt% Pd/SiC for the N-methylation reaction.

Compared with homogeneous catalysts, heterogeous catalaysts offer the advantages of easier product separation and the ability to be reused multiple times. To evaluate the cyclic stability of Pd/SiC (Figure 6), a probe reaction was conducted using 2-nitrobenzyl ether and formaldehyde solutions. The catalyst was recovered after filtration and drying. After five consecutive reaction cycles under identical conditions, the Pd/SiC catalyst exhibited no significant loss in catalytic activity or selectivity. Furthermore, TEM analysis revealed no noticeable aggregation or growth of the Pd nanoparticles, with the particle morphology remaining consistent with that of the fresh catalyst (Figure S3). XPS analysis confirmed that the Pd nanoparticles retained their metallic state after cycling (Figure S4). This indicates that the catalyst has excellent structural and chemical stability.

### 3.5. Investigation of the Reaction Mechanism

**Figure 7:**
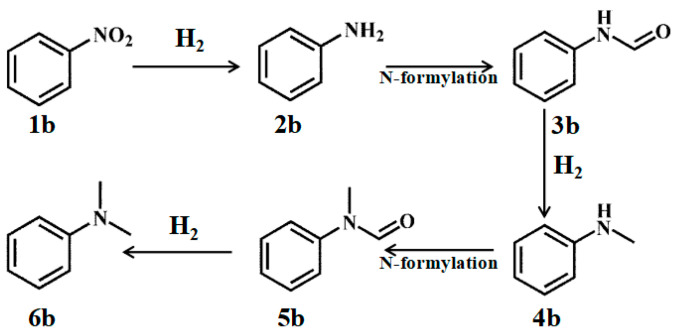
N-Methylation of nitrobenzene with formaldehyde.

Using nitrobenzene as a representative example, the typical N-methylation reaction pathway proceeds as illustrated in Figure 7. The process begins with the hydrogenation of nitrobenzene (**1b**), yielding aniline (**2b**). Aniline subsequently undergoes a carbonylation reaction with formaldehyde to form N-phenylcarbamate (**3b**), which is then hydrogenated to produce N-methylaniline (**4b**). This intermediate undergoes a second carbonylation with formaldehyde, resulting in the formation of N-methylcarbamate (**5b**). Finally, hydrogenation of N-methylcarbamate affords the target product, N,N-dimethylaniline (**6b**).

Based on the proposed reaction pathway, product distribution was investigated under identical reaction conditions using potential intermediates as substrates (Table 3). When nitrobenzene served as the substrate, only a minor amount of aniline was detected, with the primary product being the desired N,N-dimethylaniline. Notably, intermediates such as **3b**, **4b**, and **5b** were not observed. Even with a reduced reaction time of 30 min, only **4b** and **6b** were detected. When aniline was used as the substrate, **6b** was the sole product identified. Furthermore, experiments employing **3b** and **5b** as substrates showed no reaction progression. To eliminate potential interference from the formaldehyde solution, a control experiment was conducted in which **5b** was used as the substrate without the addition of formaldehyde, thereby preventing any hydrogenation reaction. These results collectively suggest that, within this catalytic system, nitrobenzene undergoes hydrogenation to aniline, which then directly condenses with formaldehyde to form the target product, bypassing any formylation steps.

**Figure 8:**
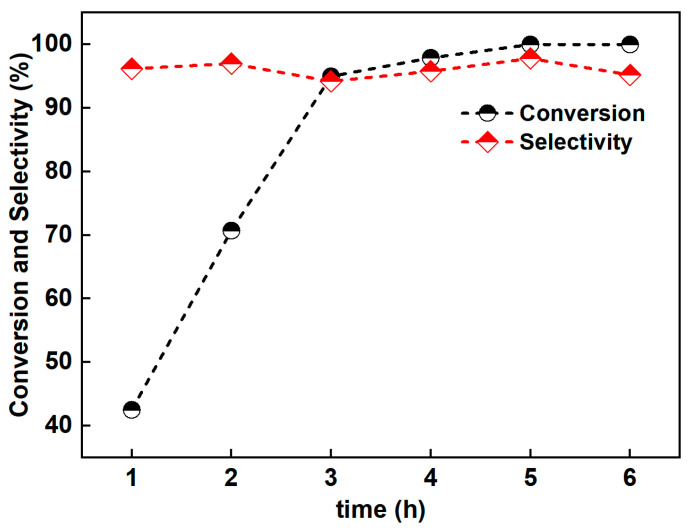
Evolution of the conversion of 2-nitroanisole and the selectivity of 2-methoxy-N,N-dimethylaniline with reaction time over the Pd/SiC catalyst.

To investigate the high selectivity of Pd/SiC, reaction kinetics experiments were conducted to examine the product distribution during the reaction (Figure 8). The results show that as time progresses, the reactants gradually convert, with 95% conversion occurring by 3 h. After 3 h, the reaction rate slows down due to the decreasing reactant concentration, and all the reactants are converted by 6 h. The selectivity of 2-methoxy-N,N-dimethyl aniline remains at about 95% from the start of the reaction at 1 h. These show that following the Pd/SiC-catalyzed nitrohydrogenation to aniline, aniline readily undergoes N-methylation with formaldehyde to yield the target product, indicating that the catalyst possesses a preferential activation capability for the N-methylation of aniline.

**Figure 9:**
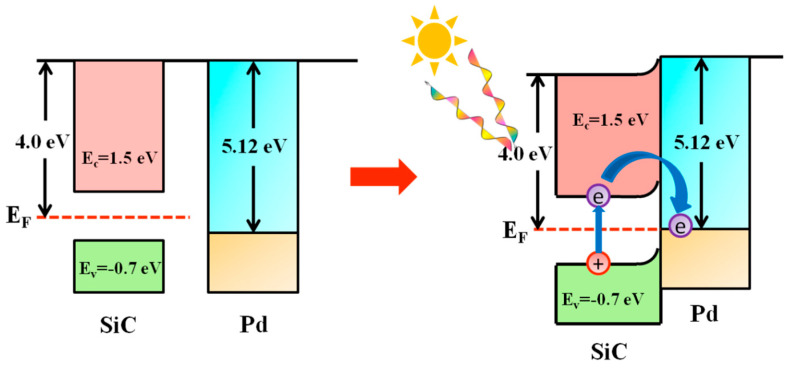
Transfer of electrons in Pd/SiC before and after illumination.

The activity of Pd/SiC is significantly greater than that of catalysts formed from Pd with other supports. To investigate the reasons, we first considered whether the morphology of Pd was different. TEM characterization was performed on three catalysts, Pd/SiC, Pd/TiO_2_, and Pd/Al_2_O_3_ (Figure S5). The average particle sizes of the three catalysts were 2.6, 2.3, and 2.5 nm, respectively, which are nearly identical. All the exposed crystal planes were Pd (111). The dispersion of metals in Pd/SiC and Pd/TiO_2_ was better than that in Pd/Al_2_O_3_, which is consistent with previous XPS results. Combining with the performance of the photocatalyst in Section 3.2, it can be concluded that the support is the key factor affecting the reaction activity, whereas the metal Pd is the primary factor influencing selectivity. This fully demonstrates the unique properties of SiC. Under light conditions, Pd can absorb visible light due to bandgap transitions, resulting in good photocatalytic activity. The Tauc plot derived from the UV-Vis-NIR spectroscopy (Figure S6) indicates that the optical bandgap of synthesized SiC is approximately 2.843 eV. It confirms the semiconductor nature of SiC support and its capability to absorb visible light up to ~500 nm. When exposed to light, photon absorption by the SiC surface promotes electrons from the valence band to the conduction band. Due to the Mott–Schottky junction formed between Pd and SiC, these conduction band electrons migrate to the Pd surface, rendering it electron-rich (Figure 9). This electron accumulation on the Pd surface enhances the dissociation of hydrogen molecules, a process identified as the rate-determining step in the reaction and a critical factor influencing overall catalytic activity. Consequently, the Pd/SiC catalyst exhibits superior performance in N-methylation reactions.

**Figure 10:**
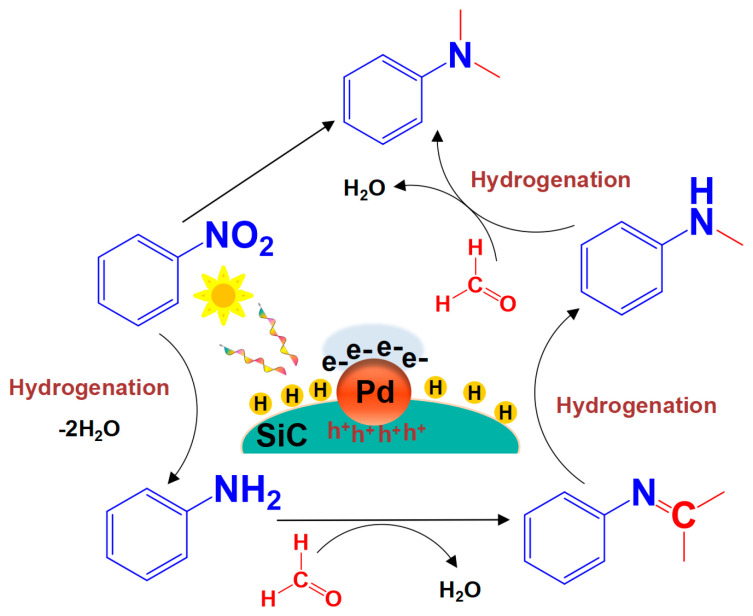
Schematic diagram of the photothermolcatalytic synthesis of N,N-dimethylaniline by one-pot nitrobenzene over a Pd/SiC catalyst.

In summary, a general explanation of the mechanism for the Pd/SiC photothermal catalytic N-methylation reaction is provided, as illustrated in Figure 10. Under light irradiation, the SiC support absorbs visible light energy, generating electron–hole pairs. Owing to the difference in work functions between Pd and SiC, an intrinsic potential is established, facilitating the transfer of electrons from the conduction band of SiC to the Pd surface. Hydrogen molecules adsorbed on the Pd surface subsequently dissociate into atomic hydrogen, which diffuses to the support surface. There, it reacts with surface-adsorbed nitrobenzene to produce aniline. Concurrently, the photogenerated electrons (e^−^) can be transferred to the formaldehyde molecule, resulting in partial protonization (reduction) of formaldehyde on the surface of Pd/SiC, thus forming a highly reactive formaldehyde intermediate. Subsequently, the amino group (-NH_2_) of aniline—generated via the hydrogenation of nitrobenzene—acts as a nucleophile and attacks the protonated formaldehyde intermediate, leading to the formation of a imine species (Ar-N = CH_2_). Thereafter, the imine intermediate is further reduced by photogenerated electrons (e^−^) derived from the Pd/SiC, producing N-methylaniline (Ar-NHCH_3_). The amino group of N-methylaniline continues to undergo a similar series of reactions with the remaining protonated formaldehyde intermediates in the system, ultimately forming N,N-dimethylaniline. This mechanism aligns with the pathway for methylation of heteroarenes via reductive amination with benzaldehyd, as reported by Chen et al. [40]. Furthermore, it aligns well with the experimental results presented in Table 3 of this work, where neither intermediate 3b nor 5b was detected, and no significant reaction occurred when either was used as the reactant.

## 4. Conclusions

In summary, Pd/SiC was synthesized via a liquid-phase reduction method and evaluated for its performance in a one-pot photocatalytic N-methylation reaction of nitro compounds with formaldehyde. Under optimized conditions—3 wt% Pd/SiC, 40 °C, 1 atm H_2_, and a light intensity of 0.25 W/cm^2^—the reaction achieved up to 100% yield of N,N-dimethylaniline. The Pd/SiC catalyst demonstrated high activity and selectivity across a range of aromatic nitro compounds bearing both electron-withdrawing and electron-donating substituents, efficiently converting them into the corresponding N,N-dimethylamine derivatives. However, substrates containing halogen substituents underwent partial dehalogenation, resulting in decreased selectivity. The intensity and wavelength of light significantly affect the activity of a reaction. A higher light intensity results in higher conversion rates and a greater contribution from light. The wavelength range of 400–450 nm has the greatest effect on the reaction. The high activity of Pd/SiC in N-methylation reactions is due to the Mott–Schottky contact between Pd and SiC, which facilitates the transfer of photogenerated electrons to Pd, placing Pd in a more electron-rich state and accelerating H_2_ dissociation. The high selectivity is attributed to the direct methylation reaction between Pd nanoparticles, which catalyzes nitro hydrogenation to amines and formaldehyde without undergoing formylation.

## Figures and Tables

**Table 1:** Photocatalytic reductive N-methylation of 2-nitroanisole using different catalysts.

Entry	Catalyst	Conv. (%)	Select. (%)	
			2-methoxy-N,N-dimethylaniline (1a)	2-methoxy-N-methylaniline (2a)
1	SiC	0	—	—
2	Pd/SiC-light	95.0	94.2	5.8
3	Pd/SiC-dark	46.1	96.4	3.6
4	Pd/SiC-Ar-light	0	—	—
5	Pt/SiC-light	96.6	57.3	42.7
6	Ru/SiC-light	9.9	81.3	18.7
7	Pd/Al_2_O_3_	14.5	90.8	9.2
8	Pd/TiO_2_	30.3	93.7	6.3
9	Pd/ZrO_2_	21.6	97.1	2.9
10	Pd/SiC (1 wt%)	72.3	85.0	15
11	Pd/SiC (5 wt%)	69.8	96.8	3.2

Reaction conditions: 0.8 mmol 2-nitrobenzyl ether, 0.13 mL formaldehyde solution, 30 mg catalyst, 7 mL ethanol, 40 °C, Xenon lamp source, 0.25 W/cm^2^, 1 atm H_2_, 3 h.

**Table 2:** Photocatalytic reductive N-methylation of different substituted nitroarenes using Pd/SiC.

Entry	Substrate	Product	Conv. (%)	Select. (%)
1	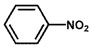	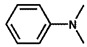	100	100
2	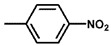	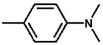	100	100
3	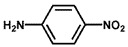	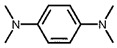	100	100
4	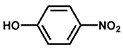	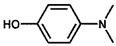	79.1	100
5	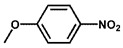	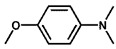	98	96.7
6	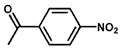	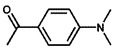	91.5	100
7	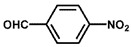	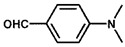	100	100
8 ^a^	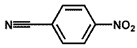	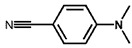	100	38.7
9	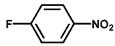	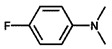	67.2	68.2
10 ^b^	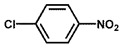	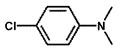	100	31.9

Reaction conditions: 0.8 mmol substrate, 0.13 mL formaldehyde solution, 30 mg Pd/SiC, 7 mL ethanol, 40 °C, xenon lamp source, 0.25 W/cm^2^, 1 atm H_2_, 3 h. ^a^ 5 h of reaction time. The by-product is N-methyl-4-aminoaniline; ^b^ The by-product is N,N-dimethyl aniline.

**Table 3:** Distribution of different substrate conversions and selectivity of the products.

Entry	Substrate	Conv. (%)	Select. (%)				
			2b	3b	4b	5b	6b
1	1b	100	<1	nd.	nd.	nd.	>99
2 ^a^	1b	61.7	<1	nd	9.3	nd	90.6
3	2b	100	—	nd.	nd.	nd.	>99
4	3b	<1	—	—	nd.	nd.	100
5	4b	100	—	—	—	nd.	100
6	5b	0.5	—	—	—	—	100
7 ^b^	5b	0	—	—	—	—	—

Reaction conditions: 0.8 mmol substrate, 0.13 mL formaldehyde solution, 30 mg Pd/SiC, 7 mL ethanol, 40 °C, xenon light source, 0.25 W/cm^2^, 1 atm H_2_, 3 h. ^a^ 0.5 h; ^b^ formaldehyde-free solution.

## Data Availability

The authors confirm that the data supporting the findings of this study are available within the article and its Supplementary Materials.

## Supplementary Materials

The following supporting information can be downloaded at: https://www.mdpi.com/article/10.3390/nano15181394/s1, Figure S1. TEM images of 3 wt% Pt/SiC and 3 wt% Ru/SiC catalysts (A,C), and the size distribution of Pt and Ru nanoparticles (B,D). Figure S2. TEM images and corresponding size distribution of Pd nanoparticles on 1 wt% (A,B) and 5 wt% Pd/SiC (C,D) catalysts. Figure S3. TEM image of the 3 wt% Pd/SiC catalyst after five reaction cycles. Figure S4. XPS spectra of the 3 wt% Pd/SiC catalyst after five reaction cycles. Figure S5. TEM images (A,C,E) and corresponding nanoparticle size distribution (B,D,F) for the 3 wt% Pd/SiC, Pd/TiO_2_, and Pd/Al_2_O_3_ catalysts, respectively. Insets in (A,C,E) display HRTEM micrographs of individual Pd nanoparticles. Figure S6. Tauc plot showing the bandgap estimation of the SiC support. The extrapolated line (blue) gives a bandgap value of 2.843 eV for an indirect transition.
