# Supplementary material for: Pd/SiC-Catalyzed Visible-Light-Driven N-Methylation of Nitroaranes Using Formaldehyde
Source: Nanomaterials (Basel). 2025 Sep 10;15(18):1394. doi: 10.3390/nano15181394 (PMC12472968; doi:10.3390/nano15181394)
Supplement: Supplementary file 1 [file nanomaterials-15-01394-s001.zip › nanomaterials-3823767-supplementary.pdf]

nanomaterials

Article

Supporting Information

Pd/SiC-Catalyzed Visible-Light-Driven N-Methylation of Nitroaranes Using Formaldehyde

Dongfang Hou [1], Ruifeng Guo [2], Xianshu Dong [2], Yuping Fan [2], Jingru Wang [2,*] and Xili Tong [3]

1. Center for Analysis Testing and Equipment sharing of TYUT, Taiyuan University of Technology, Taiyuan 030024, China
2. College of Mining Engineering, Taiyuan University of Technology, Taiyuan 030024, China
3. State Key Laboratory of Coal Conversion, Institute of Coal Chemistry, Chinese Academy of Sciences, Taiyuan 030001, China

* Correspondence: wangjingru@tyut.edu.cn

Supporting Information

Preparation of 3 wt% Pt/SiC: Pt/SiC was prepared via an in situ hydrogen reduction method. The detailed procedure is as follows: 3.9 mL of chloroplatinic acid solution (2 mg/mL), 97 mg of silicon carbide (SiC), and 0.6 g of PVP (molecular weight ~ 29,000) were dissolved in 20 mL of deionized water. The mixture was ultrasonically stirred at 500 rpm for 30 min and then transferred into a 100 mL stainless steel autoclave. Reduction was carried out under 2.0 MPa H_2 at 150 °C with stirring at 800 rpm for 2.5 h. After the reduction, the system was cooled to room temperature. The catalyst was washed three times with water and ethanol, respectively, and then dried in a vacuum oven for 12 h to obtain approximately 100 mg of the catalyst.

Preparation of 3 wt% Pt/SiC: Ru/SiC was prepared using an impregnation– reduction method. The procedure is as follows: 97 mg of SiC and 3.1 mL of ruthenium trichloride solution (2 mg/mL) were dissolved in 20 mL of ethanol. The mixture was heated at 80 °C under stirring until the solvent was completely evaporated. The resulting solid powder was then placed in a tube furnace and reduced under a 5% H_2/Ar atmosphere at 300 °C for 2 h. Finally, approximately 100 mg of the catalyst was obtained.

Apparent quantum yield (AQY) values: The incident light λ = 470 nm, the irradiation area of the reactor is 9 cm^2, and the power P = 0.25 W/cm^2 × 9 = 2.25 W. This reaction is a dimethylation reaction with electron transfer number n = 10.

Single photon energy:

$$E_{\text{photon}} = \frac{hc}{\lambda} = \frac{6.626\times10^{-34}\times3\times10^{8}}{470\times10^{-9}} \approx 4.23\times10^{-19}\,\text{J} \quad \text{(S1)}$$

Number of photons incident per unit:

$$N = \frac{P}{E_{\text{photon}}} = \frac{2.25\ \text{W}}{4.23\times10^{-19}} \approx 5.31\times10^{18} \quad \text{(S2)}$$

Total number of absorbed photons:

$$N\times t\times3600 = 5.31\times10^{18}\times3\times3600 \approx 5.73\times10^{21} \quad \text{(S3)}$$

Apparent quantum yield (AQY):

 https://doi.org/10.3390/nano15181394

$$AQY = \frac{0.8\times10^{-3}\times6.022\times10^{23}\times10}{\text{Total number of absorbed photons}}\times100\% = 8.4\% \quad \text{(S4)}$$

Figure Captions

Figure S1. TEM images of 3 wt% Pt/SiC and 3wt% Ru/SiC catalysts (A and C), and the size distribution of Pt and Ru nanoparticles (B and D).

Figure S2. TEM images and corresponding size distribution of Pd nanoparticles on 1 wt% (A, B) and 5 wt% Pd/SiC (C, D) catalysts.

Figure S3. TEM image of the 3 wt% Pd/SiC catalyst after five reaction cycles.

Figure S4. XPS spectra of the 3 wt% Pd/SiC catalyst after five reaction cycles.

Figure S5. TEM images (A, C, E) and corresponding nanoparticle size distribution (B, D, F) for the 3 wt% Pd/SiC, Pd/TiO_2, and Pd/Al_2O_3 catalysts, respectively. Insets in (A), (C), and (E) display HRTEM micrographs of individual Pd nanoparticles.

Figure S6. Tauc plot showing the bandgap estimation of the SiC support. The extrapolated line (blue) gives a bandgap value of 2.843 eV for an indirect transition.

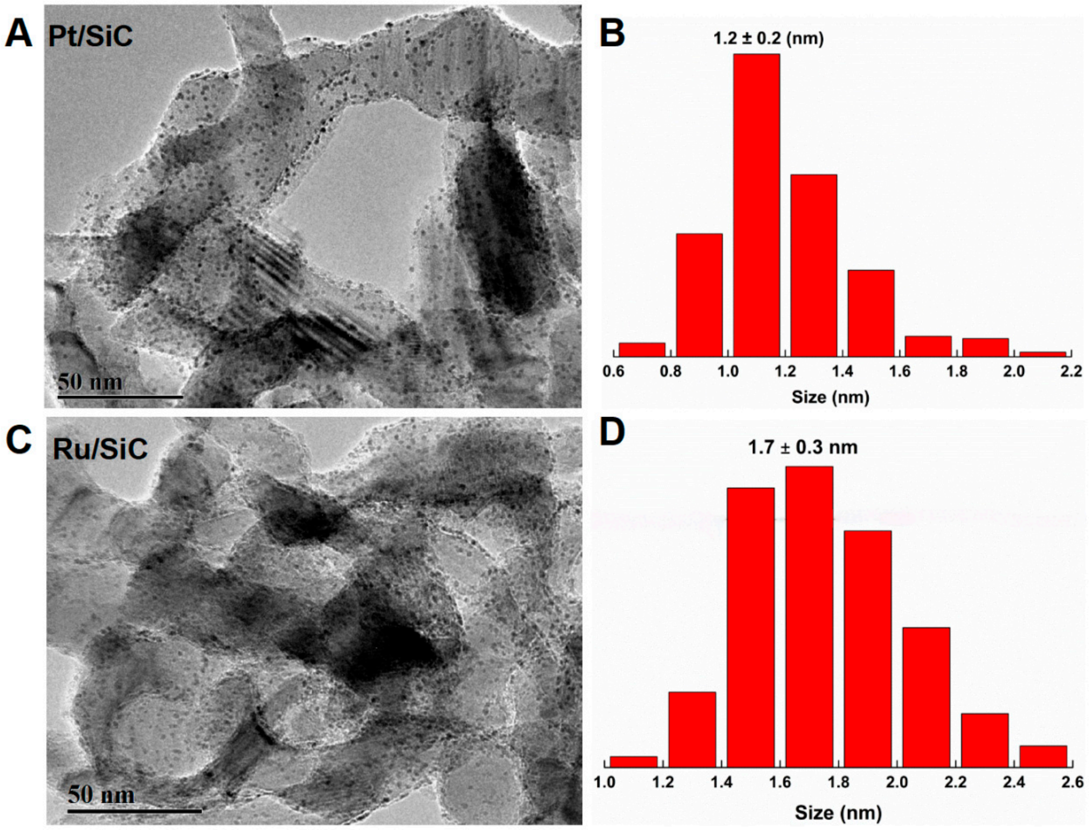

Figure S1. TEM images of 3 wt% Pt/SiC and 3 wt% Ru/SiC catalysts (A and C), and the size distribution of Pt and Ru nanoparticles (B and D).

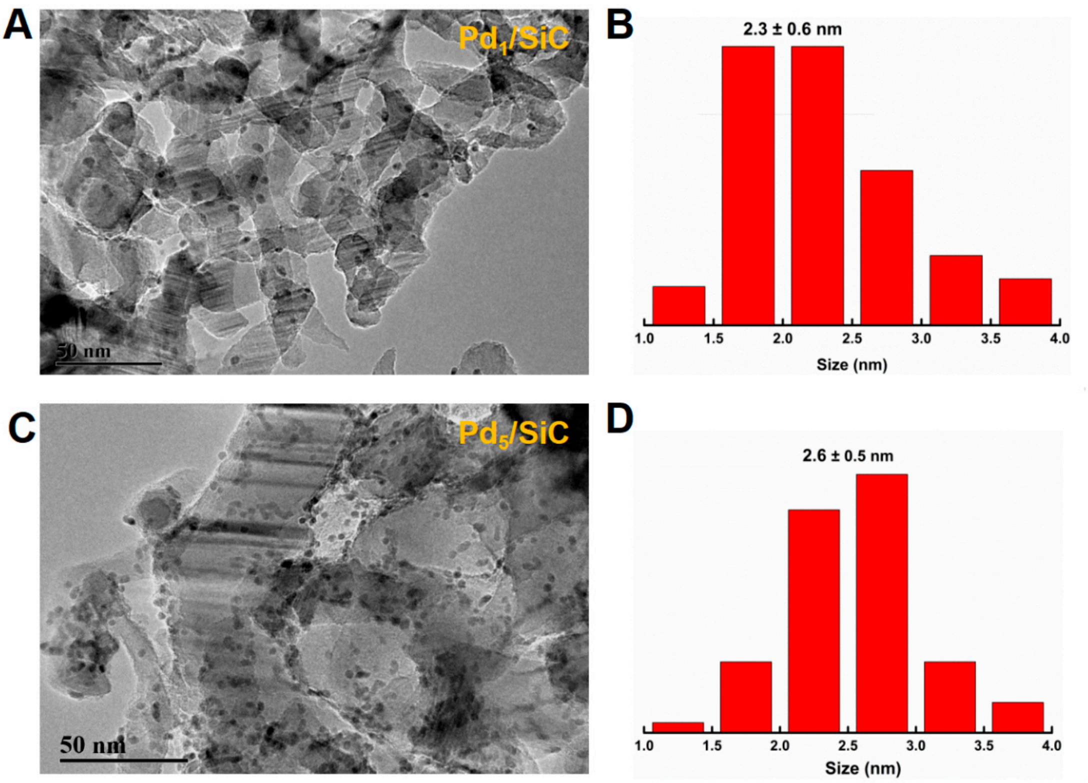

Figure S2. TEM images and corresponding size distribution of Pd nanoparticles on 1 wt% (A, B) and 5 wt% Pd/SiC (C, D) catalysts.

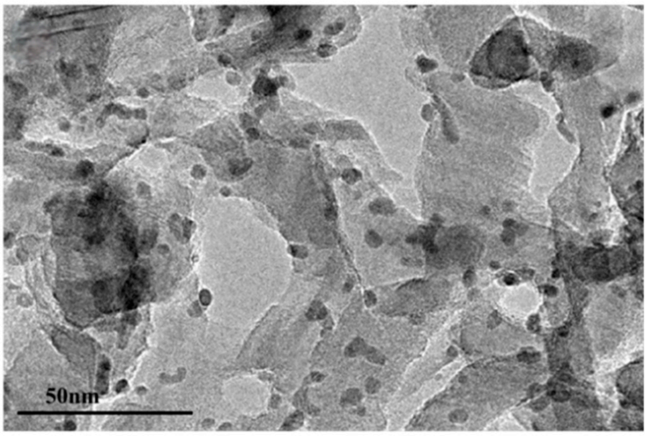

Figure S3. TEM image of the 3 wt% Pd/SiC catalyst after five reaction cycles.

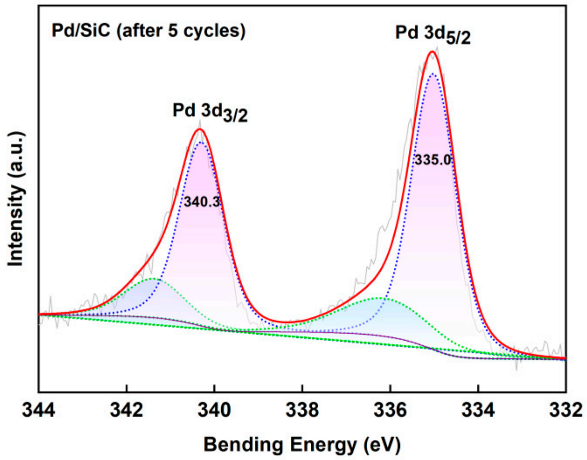

Figure S4. XPS spectra of the 3 wt% Pd/SiC catalyst after five reaction cycles.

TEM characterization of the post-reaction catalyst revealed no agglomeration or growth of the metal nanoparticles, and the morphology remained essentially unchanged (Figure S4). The XPS results of the catalyst after cycling show that the intensity of the characteristic peak of Pd $3d_{5/2}$ binding energy does not significantly decrease compared to fresh catalysts (Figure S5), and the oxidation state of Pd remains mainly metallic (Pd^0), with no obvious signal weakening due to oxidation or loss. This indicates that after multiple cycles, the palladium species on the surface of the catalyst remain stably loaded, without significant shedding.

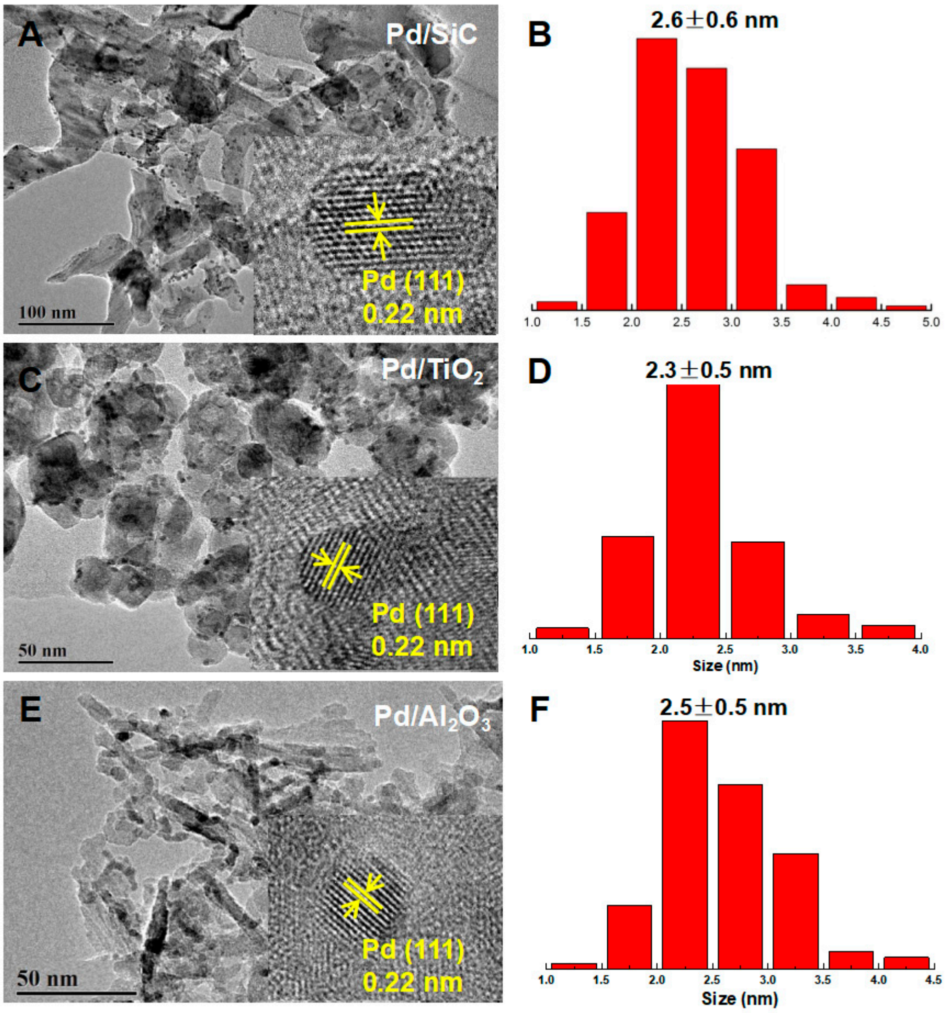

Figure S5. TEM images (A, C, E) and corresponding nanoparticle size distribution (B, D, F) for the 3 wt% Pd/SiC, Pd/TiO_2, and Pd/Al_2O_3 catalysts, respectively. Insets in (A), (C), and (E) display HRTEM micrographs of individual Pd nanoparticles.

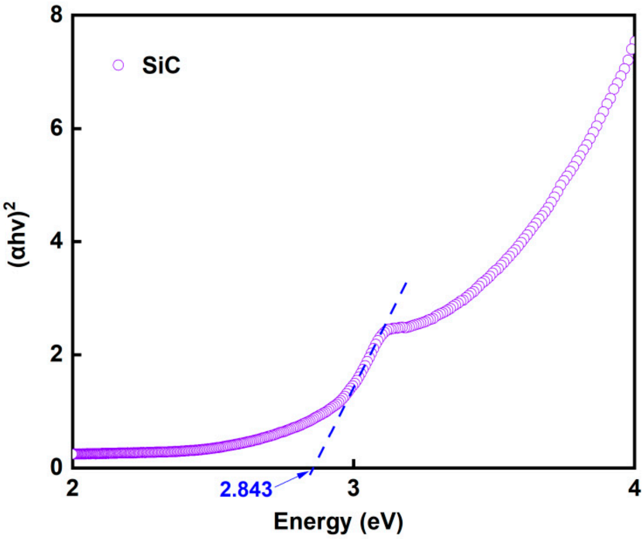

Figure S6. Tauc plot showing the bandgap estimation of the SiC support. The extrapolated line (blue) gives a bandgap value of 2.843 eV for an indirect transition.
